# Validating the self-competence in death work scale for end-of-life care volunteers

**DOI:** 10.1186/s12904-025-01666-w

**Published:** 2025-02-04

**Authors:** Zhuyun Lin, Vivian Weiqun Lou, Wallace Chi Ho Chan

**Affiliations:** 1 Faculty of Social Sciences, The University of Hong Kong, Hong Kong city, China; 2 Department of Social Work and Social Administration, The University of Hong Kong, Pokfulam Road, Hong Kong city, China; 3 Sau Po Centre on Ageing, The University of Hong Kong, Hong Kong, China; 4 Department of Social Work Education, Community Wellbeing, Northumbria University, Newcastle city, UK

**Keywords:** Self-competence, Scale validation, Volunteers, Death work, End-of-life care

## Abstract

There is an increasing demand for end-of-life care (EoLC) volunteers in Hong Kong’s aging population. However, there is no validated measure that assesses volunteers’ self-competence in coping with death. This is essential to optimize their services, and ensure their psychological well-being. This study aimed to validate the existing Self-Competence in Death Work Scale (SC-DWS) for EoLC volunteers. This scale has been tested previously on health professionals providing end-of-life care, and was adapted for this study with words that fitted the volunteer context.

A self-administered survey collected demographic information, personal experiences, the 16-item SC-DWS, 15-item Templer Death Anxiety Scale, and the 8-item Spiritual Wellbeing Scale to examine the internal consistency, concurrent validity, and discriminative validity of this scale among EoLC volunteers. The study sample consisted of all applicants who registered for the EoLC volunteer training programme between 2019 and 2021. Applicants first underwent a systematic screening and selection procedure. They completed an online registration form which included risk assessment, followed by structured individual interviews focusing on applicants’ motivation and mental preparedness for their role. The content validity of the SC-DWS was determined using data from the 341 volunteers who were screened prior to training. Construct validity was tested using Exploratory Factor Analysis (EFA), which suggested that two-factors (subscales) offered the best combination of variables (Emotional and Existential Subscales). The new subscales and the component items differed slightly from those identified in health professional samples. Concurrent validity was demonstrated by strong correlations between the SC-DWS, and the Death Anxiety, and Spiritual Wellbeing scales. Discriminant validity was supported by strong relationships between the new subscales and participants’ personal experiences. The SC-DWS was shown not only to be reliable and valid for EoLC volunteers, but it also highlighted the unique emotional challenges they faced.

## Introduction

End-of-life care for patients nearing death and their families, requires a comprehensive and coordinated approach from both medical and non-medical personnel. People receiving EoLC typically have less than 12 months to live [1]. Globally, the increasing size of the aging population has focused attention on the importance of maintaining quality of life at end-of-life (EoL) [2]. However there is a global shortage of medical professionals with expertise in providing EoLC services [3]. Consequently, volunteers have emerged as essential contributors to EoLC, by actively engaging with, and supporting professional healthcare services [4]. Worldwide, the integration of volunteers into EoLC, particularly in community settings, has been recognized as an important strategy to bridge the widening gap between increasing demand for EoLC and decreasing availability of healthcare professionals in the EoLC sector [5, 6].

The literature indicates that volunteers in EoLC have become a recognised part of healthcare systems, assuming significant and independent roles in EoLC as part of integrated multidisciplinary teams [7–11]. Such roles include offering companionship and emotional support to patients and families, providing spiritual guidance and affirmation, temporarily serving as surrogate family members, and mediating between patients, families, healthcare systems, and communities [4, 7, 8]. Volunteers’ involvement in “death work” has been defined as *“any supportive*,* therapeutic*,* or remedial work in response to death or matters related to death”*(p.900) [12]. There is evidence that volunteers’ engagement in EoLC can improve patients’ and families’ quality of life, and reduce emotional distress and death anxiety. They can also increase patients’ survival times and decrease morbidity rates [5, 10, 11] .

Nonetheless, as for professional health workers, volunteers engaged in death work can find it emotionally and physically taxing [13]. They thus require proper training to equip them for their EoLC roles. Research has shown that core competencies for volunteers are akin to those required by professional health workers, of which death work competence is integral [1, 14]. A high level of self-competence in death work can improve overall quality of EoLC and assist health workers to avoid compassion fatigue and burnout [15–17].

To date, most studies have concentrated on health professionals’ self-competence in death work, and to our knowledge, there are only two scales that measure death competence (the Coping Death Scale (CDS) and the Self-Competence in Death Work Scale (SC-DWS)). The CDS is a 30-item tool specifically created for hospice and palliative care health workers [18]. It is designed to understand health workers’ strategies in dealing with death, with eight topics about the epidemiology of death, fundamentals of human grief, crisis intervention, process of dying, community resources for prevention and coping, children and death, religious and parapsychological views of death, and organizational and societal constraints. However, the CDS does not investigate personal resources required to deal with death, and we propose that the SC-DWS addresses this gap.

Self-competence is considered as the essential skills required for health workers to effectively manage the challenges they may face in dealing with end-of-life care. These challenges include emotional aspects, such as personal grief and feelings of helplessness, as well as existential issues, which may involve questioning assumptions about life and death, and seeking meaning in life [12]. Self-competence emphasizes the importance of personal resources, which extend beyond mere knowledge and skill development [12]. In accordance with the resource congruence model [19], the coping strategies employed by an individual when faced with death are dependent on the appropriate use of personal resources. Both existential and emotional coping are seen as behavioral responses or actions taken in relation to the distress that can be experienced in death work.

Based on this perception, the 16-item SC-DWS was developed by Chan et al. in 2015 to examine the role of the self in death work, and to measure self-perceived competence of healthcare and social care professionals in dealing with death-related work [20]. The SC-DWS consists of 16 items divided into two subscales: the Existential subscale (10 items) and the Emotional subscale (4 items). Items are measured on a 5-point Likert scale. Participants are asked to rate to what extent the items are compatible with their current condition, from 1= “completely incompatible” to 5= “completely compatible”. The scale was reported as having Cronbach alpha of 0.88 for entire SC-DWS, 0.84 for Existential subscale, and 0.78 for Emotional subscale [20]. The scale ranges from 15 to 80, with a higher score indicating greater competence in coping with death.

To our knowledge, the SC-DWS has only been used in Hong Kong, and Spain, for healthcare professionals. In both contexts, validation studies have reported satisfactory psychometric properties, including good internal consistency and construct validity [1, 17, 21]– [23]. Subsequent to the development study [20], in an intervention study by Chan et al., which indicated the effectiveness of a three-day workshop in enhancing helping professionals’ self-competence in death work, SC-DWS showed good internal consistency, with Cronbach’s alpha of 0.86, 0.81 and 0.77 respectively for the complete scale, existential and emotional subscales [21]. Cheung et al. used the SC-DWS to collect data from 885 health and social care workers, reporting a mean SC-DWS score of 60.16 (range: 16–80), with Existential and Emotional subscales scoring 37.90 (range: 10–50) and 14.46 (range: 4–20), respectively. This study indicated that the SC-DWS was internally consistent (Cronbach’s alpha: 0.90 for entire scale, 0.87 for Existential subscale, and 0.79 for Emotional subscale). The SC-DWS presented a positive correlation with age and bereavement experience [22]. In the study of Vallès-Fructuoso et al., the Spanish version of SC-DWS has also shown good reliability and validity, with a Cronbach alpha 0.71 for the whole scale and significant relationships with other variables [23]. Another validation study conducted by Chan et al. indicated a significant relationship between SC-DWS and depression, which was moderated by meaning of life. The average total score for SC-DWS was 60.38, with the two subscales, existential and emotional, having respective mean scores of 38.33 and 14.03 [17].

No study has investigated the validity of SC-DWS to measure volunteers’ competence in death work. The genesis for our research was that we sought a valid and reliable measure of volunteers’ self-competence in death work, as a framework for screening and training, and providing them with appropriate supports. Unlike professional health workers, volunteers can lack systematic training or formal qualifications relevant to EoLC, and thus they may not possess specialized knowledge and skills to deal with the stresses of EoLC, or be able to draw on supports available to healthcare professionals. Furthermore, as they are unpaid, their motivation for serving people at EoL may be underpinned by strong altruistic humanitarian ambitions [5, 24], which could blur the boundaries of their involvement in death work [25] and lead to undiagnosed and untreated burnout or stress [26]. These concerns highlight the importance of determining volunteers’ self-competence in dealing with the challenges of EoLC, which may be uniquely different from that of healthcare professionals.

In response to the challenges of the aging population in Hong Kong, the Hong Kong Jockey Club End-of-Life Community Care (JCECC) project was launched in 2016. EoLC volunteers provide critical components in this initiative. Volunteer training programs deliver a well-rounded curriculum designed to cater to the diverse opportunities available to volunteers in EoLC. In collaboration with non-profit government organizations, the JCECC team at the University of Hong Kong annually recruits individuals aged 18 years and older who are interested in becoming EoLC volunteers. Applicants can register online or via paper application, and provide consent after reviewing an information sheet detailing volunteer tasks, capacity-building procedures, risks, benefits. They are asked to make a commitment to at least six months of service in the program.

Following registration, applicants complete a risk assessment and a subsequent face-to-face interview as part of a motivational screening process led by experienced EoLC social workers. This process aims to screen out potentially-unsuitable candidates for the volunteer program. Approved applicants then participate in the volunteer training program, in which they complete pretraining (T_0_), post-training (T_1_), 6-months (T_2_), and 12-month (T_3_) follow-up questionnaires. The screening and recruitment procedures, plus the questionnaires have been described elsewhere [10, 24]. Considering the crucial role of volunteers in EoLC and the lack of research on their self-competence in death work, EoLC volunteer training under JCECC provided a rare opportunity to assess whether the SC-DWS retains its psychometric properties if used for EoLC volunteers.

## Methods

### Data collection

When volunteers registered for the program, they provided demographic information. At each subsequent time point, they answered questionnaires which contained the original 16-item SC-DWS instrument, and scales measuring death anxiety and spiritual wellbeing. Data was collected over a three-year period from 2019 to 2021. The questionnaire took about 15–20 min to complete. This paper reports on analysis of the pre-training wave data (T_0_, 2019/2020/2021). This study was approved by the Research Ethics Committees of The University of Hong Kong.

### Demographic variables

These included personal age, sex, marital status, educational level, employment status, and religious beliefs.

### Self-competence in death work scale (SC-DWS)

To better accommodate EoLC volunteer context, we revised the original SC-DWS [20] wording to align with their unique situation. Specifically, items 7, 8, 10, 13, 14, 15, and 16 were modified to replace the term “work” with “EoLC volunteering,” ensuring that the scale accurately reflected the volunteer environment. The process of rephrasing the questionnaire items was carried out in collaboration with the JCECC project team, ensuring a thorough and well-considered approach to adapting the language for the volunteers.

### Death anxiety

The Templer Death Anxiety Scale (T-DAS) is a 15-item scale originally with a true–false format which was later developed to a 5-point Likert format (scored from 1 to 5, corresponding to “completely disagree” to “completely agree”). The total score of the Likert scale can range from 15 to 75, and lower scores indicate lower levels of death anxiety. The Chinese version DAS (CDAS) was translated by Wu, Tang & Kwok [27] who reported good internal consistency with true-false format. In this study we used their translation but in a 5-point Likert format. The CDAS with 5-point Likert format had been validated in the Chinese context with acceptable validity (Cronbach alpha = 0.82) [28].

### Spiritual wellbeing

The 8-item Spiritual Wellbeing subscale (SWB), which originated from the Spirituality Scale for Chinese Elders (SSCE) [29], was adopted for this study. The SSCE was originally developed and validated in its Chinese version with both elderly people in Hong Kong and Shanghai. The scale reports satisfactory consistency among the participants (alpha = 0.79 in long-term residential care sample and alpha = 0.76 in community-based care sample). The Spiritual Wellbeing subscale asks the respondents to rate the intensity of eight emotions. Responses are in the form of 5-point Likert scale, from 5= “very strong”, 4= “strong”, 3= “moderate”, 2= “slight”, and then to 1= “none”.

### Statistical analysis

Missing data were imputed using the expectation maximization (EM) algorithm. Descriptive analyses reported on the demographic characteristics of participants, and described the mean and standard deviation of SC-DWS. Exploratory Factor Analysis (EFA) examined the factor structure of the SC-DWS, utilizing principal component for extraction and direct oblimin for rotation. Reliability was assessed through evaluating internal consistency, which is crucial for ensuring a test’s validity in research. Internal consistency represents the extent to which all items within a test measure the same concept or construct, reflecting the interconnectedness of the items. Cronbach’s alpha, a measure of internal consistency, is expressed as a number between 0 and 1, with values above 0.70 generally considered acceptable [30]. This index of reliability is commonly preferred over other estimates, such as test-retest reliability, due to its ease of use and straightforward interpretation [31]. Concurrent validity was evaluated by investigating the correlations between self-competence in death work and both death anxiety and spirituality. Discriminative validity was examined by comparing the mean scores of the SC-DWS and analyzing correlations with various attributes. One way ANOVA models were tested to determine the characteristics of personal experience and its effects on volunteers’ death competence and its implications for training.

## Results

### Subjects

From 2019 to 2021, 453 volunteers were enrolled in the training program, however, only 341 responded to the pre-training questionnaire. There was minimal missing data (0.7%) which required computation.

### Demographic characteristics of participants

Table 1 lists characteristics of the 341participants. The majority were female (79.3%), half were aged between 18 and 49 years (51.1%), over half were single (54.6%), the majority had received higher education (72.7%), less than half with full-time or part-time jobs (41.1%), and two-thirds reported being affiliated with a religion (62.6%). There were no significant differences between the responses to SC-DWS related to demographic variables (sex, age, education, employment, marital status, and religion). Over half (58.4%) had no experience of looking after EoL patients, and only 19.4% and 33.2% had received training in EoLC and bereavement, respectively. The experience of caring for EoL patients significantly influenced participants’ decisions to pursue EoLC or bereavement training.

**Table 1:** Background information of participants (*N* = 341)

Demographic variables		*n*	%
Sex	Male	67	20.7
	Female	256	79.3
Age	18–29 years old	57	20.2
	30–39 years old	38	13.5
	40–49 years old	49	17.4
	50–59 years old	68	24.1
	60 or above	70	24.8
Marital status	Single	153	54.6
	Married	127	45.4
Education	Below college degree	77	27.3
	College degree and above	205	72.7
Employment status	Full-time	79	28
	Part-time	37	13.1
	Housewives	24	8.5
	Unemployed	27	9.6
	Retired	60	21.3
	Others	55	19.5
Religion	Christianity	95	33.2
	Catholicism	34	11.9
	Buddhism	42	14.7
	Traditional Chinese beliefs	4	1.4
	Others	4	1.4
	No religion	107	37.4
TCEP experience	No	164	58.4
	Yes	117	41.6
EoLC training	No	203	80.6
	Yes	49	19.4
Bereavement training	No	169	66.8
	Yes	84	33.2

Note: TCEP means “taking care of EoL patients”

### Death competence of EoLC volunteer participants

The means of the complete SC-DWS scale, and the existential and emotional subscales were 63.02 (SD = 8.08), 39.64(SD = 5.14) and 15.38(SD = 2.51) (see Table 2), all of which were higher than those reported for healthcare professionals, these being 60.16(SD = 8.39), 37.90(SD = 5.33) and 14.46(SD = 2.59) respectively [22].

**Table 2:** Correlations among variables

	Mean (SD)	1	2	3	4	5	6	7	8	9	10	11
1 SCDWS_ Existential _new	23.68(3.54)	1										
2 SCDWS_ Emotional _new	27.38(3.89)	0.58**	1									
3 SCDWS _new (13 items)	51.07(6.61)	0.88**	0.90**	1								
4 SCDWS_ Existential	39.64(5.14)	0.93**	0.82**	0.98**	1							
5 SCDWS_ Emotional	15.38(2.51)	0.57**	0.87**	0.82**	0.69**	1						
6 SCDWS_S (16 items)	63.02(8.08)	0.85**	0.91**	0.99**	0.96**	0.86**	1					
7 DAS_T0_S	39.46(7.65)	− 0.50**	− 0.38**	− 0.49**	− 0.49**	− 0.41**	− 0.48**	1				
8 SWB	34.47(4.14)	0.40**	0.33**	0.41**	0.41**	0.34**	0.41**	− 0.37**	1			
9 TCEP experience		0.19**	0.15**	0.20**	0.20**	0.15**	0.20**	− 0.15*	0.09	1		
10 EoLC training		0.15*	0.17**	0.18**	0.17**	0.17**	0.19**	-0.07	0.06	0.39**	1	
11 Bereavement training		0.13*	0.15*	0.16*	0.14*	0.18**	0.16*	-0.05	0.03	0.21**	0.44**	1

Note: ** Correlation is significant at the 0.01 level (2-tailed)

* Correlation is significant at the 0.05 level (2-tailed)

Note: TCEP means “taking care of EoL patients”

### Internal consistency of SC-DWS in EoLC volunteer

The complete SC-DWS and the two subscales were all internally consistent, with Cronbach’s alpha 0.90 for the entire scale (16 items) and 0.83 for existential subscale (10 items) and 0.78 (4 items) for the emotional subscale, indicating a good internal consistency in the volunteer sample.

Adhering to the original validation study for healthcare professionals, a 2-factor solution was adopted to run the EFA by excluding items 13 and 15. Lower factor loading was found in item 9 (less than 0.4), and after eliminating this, a 13-item scale accounted for 53.60% of the total variance. The final factor analysis table is displayed below (Table 3).

**Table 3:** Factor structure of SCDWS in volunteer sample

Items	Emotional	Existential
SC-DWS1 [I can fully accept that I cannot completely control life, for example, the life and death of a patient.]		0.729
SC-DWS2 [I can fully accept that suffering is inevitable, for example, the suffering of a patient during the dying process.]		0.733
SC-DWS3 [I am prepared for my death and that of my family, for example, being open to discuss death with my family, or think about my funeral.]		0.724
SC-DWS4 [I have finished most of my business, which has reduced my life regrets.]		0.665
SC-DWS5 [Realizing that no one has full control over matters of life and death has increased my appreciation for life.]		0.515
SC-DWS6 [Realizing that no one has full control over matters of life and death, I live my life more positively and have found my meaning in life.]		0.470
SC-DWS7 [I can fully accept that my emotions are aroused during work.]	0.624	
SC-DWS8 [I can effectively cope with my emotions induced by work.]	0.776	
SC-DWS9 [I have coped with my bereavement experience or experience related to death.]	removed	
SC-DWS10 [If I feel stressed by work, I can take care of my own needs properly.]	0.828	
SC-DWS11 [I can fully accept the nature of death work, including pity or depressed feelings.]	0.776	
SC-DWS12 [Even though death is inevitable, I admire my contribution to work.]	0.707	
SC-DWS14 [I do not bring work-induced emotions into life and do not bring life-induced emotions into work.]	0.681	
SC-DWS16 [I can fully accept that, even though I am a helping professional, I feel helpless about life and death.]	0.597	

The 2-factor solution was similar to that found for health professionals but with different item number distribution for each subscale: six for the existential subscale and seven for the emotional subscale. This could be explained according to the original concept of death work competence, which included personal resources and coping strategy [12]. The Cronbach’s alpha of new subscale existential (6 items) is 0.79 and 0.85 for the new subscale of emotional (7 items), indicating good reliability for the two subscales of 13-item SC-DWS in volunteer’s sample.

### Concurrent validity

As displayed in Table 2, there were significant correlations with death anxiety scale and spiritual wellbeing scale, which supported the concurrent validity of 13-item SC-DWS in volunteers.

### Discriminative validity

Correlation analysis in Table 2 indicated that experience of taking care of EoL patients was significantly associated with the whole scale (*r* = 0.20, *p* < 0.01), the 6-item existential subscale (*r* = 0.19, *p* < 0.001) and 7-item emotional subscale (*r* = 0.15, *p* < 0.01). The experience of receiving training in EoLC and bereavement shared similar results, indicating good discriminative validity of 13-item SC-DWS in volunteers.

### Mean score of 13-item SC-DWS for participants with different personal experience

We grouped participants’ personal experiences into four categories: (1) those without any experience or training in taking care of EoL patients; (2) those without experience of taking care of EoL patients but who had received training; (3) those with experience of taking care of EoL patients but had not received training; and (4) those with experiences of taking care of EoL patients and had received training. The ANOVA findings (Table 4) showed that EoLC experience and training significantly affected self-competence in death work. Experience in taking care of EoL patients and receiving training improved participants’ self-competence in death work. Notably, individuals without any experience in taking care of EoL patients or who had not received training had the lowest scores, while those with both experiences and training had the highest scores. In the new existential subscale, having experience in caring for EoL patients and receiving training significantly differed from not having any experience in EoL care, irrespective of training status. The revised emotional subscale demonstrated significant differences only between individuals who had both experience and training, and those who lacked any experience. No differences were observed between individuals with both experience and training, and those who had either experience or training alone.

**Table 4:** SC-DWS by personal experiences (4 groups)

	Personal experience	*n*	Mean	SD	F	*p*
Existential_new	no TCEP experience and no training ^a*^	124	23.06	3.00	6.22	< 0.001
	no TCEP experience but with training ^b*^	40	22.63	3.65		
	with TCEP but no training	61	23.51	3.74		
	with TCEP and with training ^a* b*^	56	25.13	3.15		
	Total	281	23.51	3.39		
Emotional_new	no TCEP experience and no training ^a*^	124	26.84	3.77	4.18	0.006
	no TCEP experience but with training	40	27.20	4.01		
	with TCEP but no training	61	27.34	3.86		
	with TCEP and with training ^a*^	56	28.93	3.07		
	Total	281	27.42	3.76		
SCDWS (13 items)	no TCEP experience and no training ^a*^	124	49.90	6.04	6.45	< 0.001
	no TCEP experience but with training ^b*^	40	49.83	7.08		
	with TCEP but no training ^c*^	61	50.85	6.59		
	with TCEP and with training ^a* b*c*^	56	54.05	5.00		
	Total	281	50.92	6.31		
SCDWS (16 items)	no TCEP experience and no training ^a*^	124	61.54	7.53	6.38	< 0.001
	no TCEP experience but with training ^b*^	40	61.65	8.72		
	with TCEP but no training ^c*^	61	62.92	7.95		
	with TCEP and with training ^a* b*c*^	56	66.67	6.05		
	Total	281	62.88	7.75		

Note: “a* & b* c*” means significance between the two items

## Discussion

This paper reports on new information which suggests that the Self-Competence in Death Work Scale is valid for EoLC volunteers in Hong Kong. The findings suggest that the SC-DWS used on a volunteer sample exhibits satisfactory reliability and validity, not only for the entire scale but also for its two subscales. Concurrent validity and discriminability were supported, and the two-factor solution was maintained. The volunteer application of the SC-DWS scale consists of the same two subscales as the original healthcare professional scale, albeit with different items, and numbers of items (existential (6 items) and emotional (7 items)). Thus, the volunteer version of the scale reflected the distinct concerns of EoLC volunteers.

The overall scores of volunteers on the scale was significantly higher than those of the professional groups reported in Cheung et al.’s sample [22]. It is possible that volunteers might have overestimated their self-competence in dealing with death-related work, while health professionals, with their more extensive training and experience, could be more discerning in evaluating their own competence. However, we cannot rule out the possibility that these new volunteers, due to their rich life experiences, may have accumulated substantial personal resources and capabilities in handling death-related work. Factors such as age, work experience, and exposure to bereavement can all impact on their competence in death work [22]. Individuals with prior bereavement experiences tend to have higher abilities in dealing with death-related situations. Many volunteers who participate in EoLC services do so, because they have had experiences caring for terminally ill family members [32], which may also contribute to their higher competence in this area. In addition to the differences in overall scores, the disparities between the two subscales also led us to speculate that the differences between volunteers and professionals (in terms of experience and training levels) might result in some variations in the scale structure. These variations could potentially lead to inconsistencies in the scale scores.

Specifically, the emotional subscale has been changed in the volunteer version of SC-DWS. Item 9 was removed due to low loadings, while items 7, 11, 12, and 16 were newly included. Items 7 and 11 pertain to acceptance of emotions and the nature of volunteer work in EoLC. The reason for these two items loading on the emotional subscale may reflect volunteers’ concerns, and emphasis on the emotional impact of acceptance in doing volunteer work in EoLC. In fact, researches have also shown that volunteers may often experience heightened negative emotions when discussing death-related topics [33–35]. Difficulties in accepting their emotions in the volunteer work of EoLC and its nature may have a large emotional impact on volunteers, such as death anxiety. Death anxiety has been found to influence volunteering and can lead to withdrawal [11, 14]. Consequently, training in death acceptance is essential for volunteers in managing their emotional responses [13]. When compared with volunteers, healthcare professionals may perceive acceptance of emotions and nature of death work being less impactful on their emotions as a result of their professional training, but they are more concerned about how they cope with these challenges to maintain the meaning of their jobs [36].

Items 12 and 16 are about the contribution, and helpfulness of volunteer work in EoLC. These two items were loaded on the emotional subscale, which may indicate that volunteers perceive their contribution and being helpful as impacting more on their emotions. Volunteers in EoLC may often be passionate about and committed to helping, but the consequence is that they may feel emotionally challenged if they fail to contribute and help in their volunteer work. Studies have indicated that not feeling ‘good enough’ or experiencing a sense of guilt or anxiety that they were not doing enough may become volunteers’ emotional burdens [25, 37]. In contrast, healthcare professionals may focus more on the meaning of their work, which correlates with the existential dimension. When healthcare professionals fail to help, although they may still be emotionally affected, they may consider it more challenging to the existential domain which is directly related to their choice of career in working with death and bereavement [38]. The differences in the loadings of items to emotional and existential subscales of SC-DWS therefore may reflect the different concerns between volunteers and helping professionals. It seems that volunteers may particularly experience more emotional concerns, and our findings thus shed light on how training could be provided to volunteers for enhancing their competence in undertaking death work.

Additionally, our findings highlight the significance of EoL caregiving experiences and relevant training for volunteers. Individuals with both caregiving experience and training display the greatest self-competence in death work. We discovered that, for the existential subscale, having experience is crucial while training makes no difference in this aspect. However, for the emotional subscale, either experience or training can contribute to emotional preparedness. Thus, it is essential for service agencies to screen volunteers and provide specific training for those admitted, particularly for individuals without any prior experience or training in end-of-life care for their emotional coping strategies when confronting with death. Specific training methods, such as life reflective diaries, can help establish service boundaries and promote self-care among volunteers [39].

### Limitations

This research has several limitations. Firstly, a potential limitation is the reliance on self-reported data from the volunteers, which might be subject to social desirability bias or recall bias. This limitation might impact on the generalizability of the findings and the overall validity of the SC-DWS scale for EoLC volunteers. Future research could benefit from testing on other volunteer samples, as well as incorporating additional data sources, such as observational data or interviews with supervisors, to cross-validate the findings and strengthen the conclusions. Secondly, to better understand the implications of higher SC-DWS scores in the EoLC volunteer context, qualitative studies could be conducted to explore volunteers’ experiences and the specific competencies they acquire from personal exposure to death work. This in-depth exploration may also contribute to refining the construct of self-competence in death work, as well as providing insights into the practical applications of the scale in volunteer training and support. A test-retest reliability study could be conducted to enhance understanding of how the scale works over time. By addressing these limitations in future research, understanding of self-competence in death work among volunteers can be further refined, and the applicability of the SC-DWS can be expanded to enhance volunteer training and support across a range of contexts.

## Conclusion

The revised scale offers valuable insights into the specific concerns and needs of EoLC volunteers. The validation of the SC-DWS for a volunteer population contributes to the growing body of literature on volunteer self-competence in death work. It has the potential to inform and improve future recruitment, training, and support for EoLC volunteers.

## Data Availability

The dataset analyzed during the current study is available from the corresponding author on reasonable request.
